# FDA-Regulated AI-Enabled Medical Devices With Pediatric Indications

**DOI:** 10.1001/jamanetworkopen.2026.2636

**Published:** 2026-03-20

**Authors:** Grzegorz Zapotoczny, Ansh Goyal, Madison Christmas, Shahida Qazi, Michael Carroll, Juan Espinoza

**Affiliations:** 1 Stanley Manne Children’s Research Institute, Ann & Robert H. Lurie Children’s Hospital of Chicago, Chicago, Illinois; 2 Department of Surgery, University of Colorado Anschutz Medical Campus, Aurora

## Abstract

**Importance:**

Artificial intelligence (AI)–based technologies hold promise for faster and more accurate devices in health care; however, little is known about their availability to pediatric patients. A comprehensive analysis of US Food and Drug Administration (FDA) regulatory submissions is necessary to identify technologies with pediatric labeling.

**Objective:**

To characterize AI devices marketed in the US, identify those with pediatric indications, and provide clues on AI-specific barriers to pediatric device innovation.

**Design, Setting, and Participants:**

This retrospective descriptive cross-sectional study was conducted using public data from the FDA’s AI-Enabled Medical Device List. Marketing submissions reviewed by the FDA between November 1995 and June 2024 were analyzed.

**Main Outcomes and Measures:**

Prevalence of pediatric AI devices and their main characteristics (eg, clinical area, review time, and year of FDA marketing decision).

**Results:**

Among 952 submissions, 42 (4.4%) included pediatric age ranges (0-17 years). The first pediatric-inclusive device was cleared in 2015, and 5 exclusively pediatric technologies were introduced between 2020 and 2024. Of 18 clinical areas, radiology comprised 723 of all devices (75.9%) but only 18 of the devices specifically labeled for pediatrics (42.9%), whereas neurology comprised 34 devices overall (3.6%) vs 13 pediatric devices (31.0%); 10 clinical areas (55.6%) were missing among pediatric devices. The median (IQR) FDA review time was significantly longer for pediatric than for nonpediatric devices (162 [114-228] days; 95% CI, 151-212 days vs 134 [87-214] days; 95% CI, 149-162 days; 2-sided Mann-Whitney *U* test *P* = .049). Based on National Clinical Trial Identifiers in FDA summaries, clinical trial registration was noted in 6 pediatric (14.3%) vs 20 of 906 nonpediatric (2.2%) submissions.

**Conclusions and Relevance:**

In this study, pediatric devices were rare, emerged recently, and had longer review times and a higher proportion of registered clinical trials compared with nonpediatric devices, suggesting expectations for more pediatric-specific evidence despite unchanged statutory standards. To address gaps in pediatric device development, the FDA should standardize age labeling and validation requirements for AI-enabled technologies.

## Key Points

**Question:**

What proportion of marketing applications for artificial intelligence (AI)–enabled medical devices reviewed by the US Food and Drug Administration (FDA) from 1995 to 2024 had pediatric indications?

**Findings:**

In this cross-sectional analysis of 952 regulatory submissions between 1995 and 2024, few devices were specifically labeled for children (ages 0-17 years); fewer still were exclusively pediatric, with the first one authorized for marketing in 2020. The top 3 pediatric clinical areas were radiology, neurology, and cardiology, with no pediatric-specific devices in most areas.

**Meaning:**

These findings indicate that despite 30 years of AI devices on the market, pediatric-specific technologies emerged recently and remain scarce.

## Teaser

This cross-sectional study investigates the proportion of artificial intelligence (AI)–enabled devices regulated by the US Food and Drug Administration (FDA) that had pediatric indications.

## Introduction

The application of artificial intelligence (AI) in health care has been of great interest to the research community as evidenced by numerous recent publications.^1,2,3,4,5,6^ According to the US Food and Drug Administration (FDA) database, AI-enabled devices have been marketed in the US since 1995.^7^ We aimed to summarize the 30 years of AI innovation with a focus on pediatrics.

In the US, the Center for Devices and Radiological Health (CDRH) at the FDA regulates devices under a risk-based framework (class I-III). Depending on a device’s risk and novelty, it may be marketed via premarket notification, or more commonly 510(k); de novo classification; or premarket approval (PMA). Notably, many class I and some class II devices are exempt from premarket review, and a small subset use alternative routes (eg, humanitarian device exemption).^8^ AI use in medical devices presents unique regulatory challenges; for example, if an algorithm continues to learn and change over time, it could diverge from the initial, FDA-reviewed version, raising concerns about safety and continued effectiveness. The challenges of digital technologies, like cybersecurity, privacy, and quality controls, led to the creation of the Digital Health Center of Excellence (DHCoE) in 2020.^9^ The DHCoE is housed within CDRH but is not involved in marketing authorization decisions; rather, it aligns and coordinates digital health work across the FDA. Shortly after creating DHCoE and in response to the previously solicited feedback,^10^ the FDA released a plan entitled “Artificial Intelligence/Machine Learning (AI/ML)-Based Software as a Medical Device (SaMD) Action Plan.”^11^ The document introduced the concept of predetermined change control plans for AI-enabled devices, which the FDA followed on with 2 recently published draft guidance documents.^12,13^

As of November 2024, there have been 952 entries in the FDA AI device database. The availability of AI technologies for pediatric patients cannot be readily ascertained from this or any other FDA database given the lack of pediatric-specific information gathered and published during the regulatory process.^14^ The scarcity of pediatric medical devices and the issues with inclusion of pediatric patients in device studies are well documented,^15,16,17,18,19,20,21,22^ as are the numerous ethical, regulatory, clinical, engineering, and financial barriers that hinder the development of pediatric technologies.^8^ As a result, pediatric medical devices tend to lack in sophistication and availability, often lagging behind adult counterparts by as much as a decade.^21^ We aimed to characterize the extent to which FDA-regulated AI-enabled devices included pediatric evaluation and pediatric indications in their labeling. By linking pediatric labeling analysis to existing AI device regulatory frameworks, we documented emerging gaps in the availability of AI technologies to inform pediatric care, highlighted existing medical devices that may address unmet clinical needs in pediatric patients, and proposed policy changes to enhance the availability of AI-enabled medical technologies to all patients.

## Methods

This cross-sectional study used publicly available information on medical devices. As research not involving human participants, it did not require institutional review board review per the Common Rule.

### Data Sources

The analysis included all 952 AI devices listed in the publicly available FDA AI-enabled medical devices database^7^ as of November 2024. Matching device regulatory submission documents were used to extract additional information on each device from the FDA 510(k), de novo, and PMA databases. We excluded 4 devices from analysis because they did not have an indications for use statement available; therefore, 948 devices was the number of devices used for labeling pattern analysis.

### Data Extraction and Validation

A custom natural language processing program was developed to automate extraction of indications for use statements from every summary or approval order statement PDF file. Programmatically extracted PDF text was sent to gpt-3.5-turbo-0125 (OpenAI) with a prompt to extract relevant free text; the output was human validated. Other data were manually extracted from FDA databases and entered into an online database (Airtable). G.Z. and J.E. were responsible for team training and performing quality checks on extracted data elements to ensure accuracy. To identify devices indicated for pediatric patients, age labeling practices were analyzed as described in Zapotoczny et al.^23^ Briefly, we analyzed indications for use statements for numerical age ranges or descriptive, concept-based terms. When these were present, we defined *pediatric* as ages 0 to 17 years (inclusive) and *young adult* as ages 18 to 21 years (inclusive) to mirror the customary transition to adulthood at age 18 years. This differs from the CDRH definition of pediatrics, which is birth up to age 22 years.^24^ We used the FDA review, advising panel, or committee name or regulation medical specialty as listed in FDA databases to infer the device’s clinical domain. Sponsor names were transcribed from FDA databases and normalized to account for naming variations. The sponsor country of origin was selected based on the location of the global corporate headquarters.

### Statistical Analysis

Descriptive statistical analysis was performed using GraphPad Prism 10 version 10.5.0 (GraphPad Software). Group analysis was performed using the Kruskal-Wallis test with Dunn multiple comparisons. Pairwise comparisons were conducted using a 2-sided Mann-Whitney U test. Statistical significance was set at *P* < .05.

## Results

### Overview of AI-Enabled Device Landscape, 1995 to 2024

**Figure 1.**
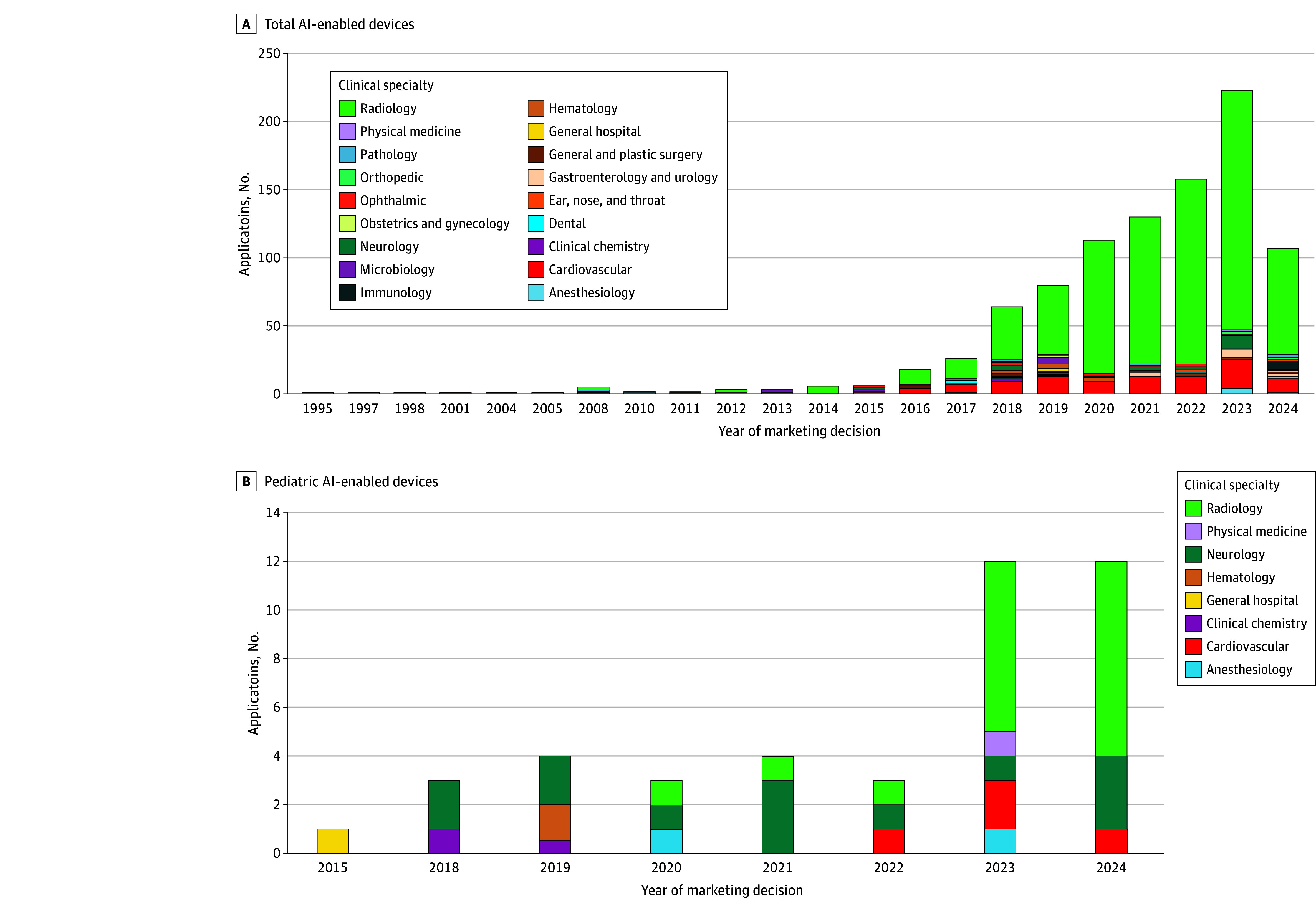
Bar Chart of Artificial Intelligence (AI)–Enabled Devices by Year

The first AI device identified in the FDA database was approved via PMA in 1995. Although the subsequent 20 years brought only 32 devices, every year since 2016 yielded a record number of new devices, with a total of 952 devices (Figure 1). This trend corresponds with the increasing number of AI device sponsors; we found only 23 between 1995 and 2016, which constitutes 6.2% of all 372 sponsors identified as of June 2024. Most FDA marketing applications for AI devices were 510(k)s (925 applications [97.2%]), with only 4 devices approved via PMA and 23 marketed through the de novo pathway (Table 1). The median (IQR) time to review 510(k) applications was 134 (87-210) days (95% CI, 147-160 days), which was significantly faster (Kruskal-Wallis test) than de novo applications (median [IQR] time, 264 [149-364] days; 95% CI [207-331] days; *P* < .001) and PMAs (median [IQR], 349 [IRQ] 230-398 days; 95% CI, 176-476 days; *P* = .02). Given that the 510(k) pathway is used for devices that are neither novel nor high-risk, these results are consistent with the notion that a known device without AI functionality was used as a predicate. Despite this observation, the review time greatly varied across 510(k) applications, from 7 to 722 days, as indicated by FDA records.

**Table 1.** Characteristics of Artificial Intelligence–Enabled Devices

Characteristic	Entries, No. (%) (N = 952)	Review time, median (IQR), d
Application type		
PMA	4 (0.4)	349 (230-398)
De Novo	23 (2.4)	264 (149-364)
PMN or 510(k)	925 (97.2)	134 (87-210)
Clinical area		
Radiology	723 (75.9)	127 (84-197)
Cardiology	99 (10.4)	153 (107-266)
Neurology	34 (3.6)	149 (103-203)
Hematology	18 (1.9)	245 (135-316)
Gastroenterology and urology	14 (1.5)	129 (29-253)
Ophthalmology	10 (1.1)	162 (88-226)
Anesthesiology	9 (0.9)	275 (133-433)
Pathology	8 (0.8)	260 (98-269)
Clinical chemistry	8 (0.8)	285 (178-342)
Microbiology	6 (0.6)	236 (201-288)
General and plastic surgery	6 (0.6)	146 (70-199)
Orthopedics	5 (0.5)	237 (132-258)
General hospital	4 (0.4)	200 (117-279)
Dentistry	3 (0.3)	217 (185-269)
Ear, nose, and throat	2 (0.2)	117 (98-135)
Physical medicine	1 (0.1)	89 (NA)
Obstetrics and gynecology	1 (0.1)	290 (NA)
Immunology	1 (0.1)	328 (NA)

Abbreviations: NA, not applicable; PMA, premarket approval; PMN, premarket notification.

### Radiological Devices Dominate the AI Space

Of the 952 AI devices, 723 devices (75.9%) were developed for applications in radiology (Table 1). FDA product codes can be used to identify device groups. Radiologic devices encompassed 27 different product codes, with the most prevalent ones related to radiological image processing (codes LLZ and QIH; 268 devices), x-ray tomography (code JAK; 73 devices), and ultrasonic pulsed doppler imaging (code IYN; 54 devices). Other well-represented clinical domains were cardiovascular (99 devices [10.4%]), neurology (34 devices [3.6%]), hematology (18 devices [1.9%]), and gastroenterology and urology (14 devices [1.5%]). The remaining clinical specialties featured 10 or fewer devices each (Table 1).

### Dichotomies in AI-Enabled Device Sponsor Profiles

The analysis of device sponsors who received FDA decisions revealed that of 372 unique applicants, 228 applicants (61.3%) had only 1 product on the market and 335 applicants (90.1%) had fewer than 5 devices. However, the top 6 companies, with 21 to 68 devices each (GE Healthcare, Siemens, Canon Medical Systems, Aidoc Medical, Philips Healthcare, and Shanghai United Imaging Healthcare), still accounted for nearly one-quarter of all AI devices (233 devices [24.5%]) (eTable in Supplement 1). Geographically, 467 devices (49.1%) were developed by companies registered in 33 US states, with California home to 117 devices (12.3%). Among international contributors, the top 5 countries (Israel, France, South Korea, Japan, and China) were home to companies responsible for 286 devices (30.0% of all devices) (eTable in Supplement 1).

### Omission of Age in Indications for Use Statements

To better understand the type of patient populations AI devices were developed for, we analyzed indications for use statements for inclusion of age descriptors. We found that 565 of 948 AI devices with indications for use statements available (59.6%) were silent on age (Table 2). This means the information was limited to device function or a medical condition the device addressed, without specifying intended patient population ages. Among 383 statements containing age information, we characterized labeling patterns to help determine intended pediatric patient populations (Table 2). The most prevalent pattern (224 statements [58.5%]) was the use of descriptive language to indicate broad age ranges (eg, *adult patients* or *patients of all ages*). Only 22 devices used discrete numbers for lower and upper bounds of the indication, while 134 devices followed a discrete number with a phrase (eg, *and older* or *and younger),*

**Table 2.** Age Labeling Patterns Across Indications for Use Statements^a^

Characteristic	Device statements, No. (N = 948)^b^	Example
Lower bound		
Structured (discrete No.)	156	“2 years old”; “18 years old”
Unstructured (concept)	0	“younger than”; “and below”
Upper bound		
Structured (discrete No.)	22	“72 months”; “16 years”
Unstructured (concept)	134	“and older”; “and above”
Labeling variation patterns		
Fully structured age range	22	“0 - 12 months”; “5 - 67 years old”
Partially structured age range	134	“above the age of 40”; “18 years and older”
Broad age range	224	“adult patients”; “all ages”
Narrow age range	3	“fetal”; “newborns”
Silent on age	565	NA

^a^ Age inclusion information among artificial intelligence–enabled devices is presented.

^b^ Data summarize 948 device labeling statements; 4 devices in the database did not contain labeling documentation.

### Scarcity of AI-Enabled Devices for Pediatric Patients

**Figure 2.**
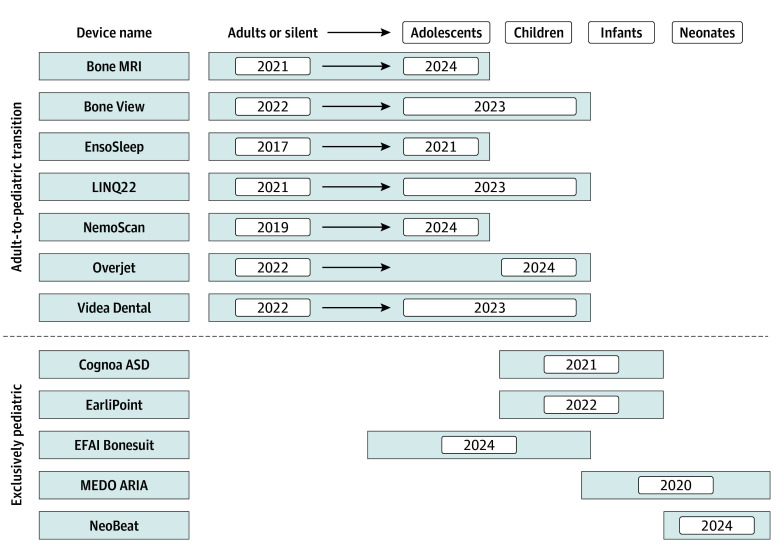
Timeline of Exclusively Pediatric Devices and Pediatric Labeling Expansions This graphical representation of the emergence of pediatric medical devices is shown through the adult-to-pediatric transition of the initial device (top) or through deliberate pediatric design (bottom). There were 7 exclusively pediatric device submissions identified; 2 submissions included a modification of an existing device. Therefore, 5 unique pediatric technologies are visualized and reported on. The year of each Food and Drug Administration marketing decision for a given labeling indication is given. MRI indicates magnetic resonance imaging.

Of 116 devices with indications that included patients younger than 22 years, 15 devices (12.9%) followed the FDA-recommended pediatric subpopulations age ranges and 74 devices (63.8%) were labeled specifically for young adults (ages ≥18 years). A total of 42 devices (4.4%) were labeled for pediatric patients aged 0 to 17 years; the first such device was marketed in 2015. Among them, 5 unique devices were exclusively pediatric (Figure 2). In terms of the breadth of pediatric submissions based on the FDA regulation medical specialty and review panels, the 42 pediatric devices identified in this study represented 8 of 18 clinical areas (44.4%). The most represented areas were radiology (18 devices [42.9%), neurology (13 devices [31.0%]), and cardiovascular (14 devices [9.5%]). Notable areas, such as gastroenterology and urology, pathology, dentistry, and ophthalmology, lacked device representation, with a total of 10 clinical areas (55.6%) having no pediatric devices. The median (IQR) FDA review time for 42 pediatric devices (162 [114-228] days; 95% CI, 151-212 days) was significantly longer than for 906 nonpediatric, non-PMA devices (134 [87-214] days; 95% CI, 149-162 days; 2-sided Mann-Whitney *U* test *P* = .049). There was National Clinical Trial (NCT) registration record for 6 pediatric (14.3%) vs 20 nonpediatric (2.2%) submissions. A detailed comparison between characteristics of AI devices with pediatric and nonpediatric indications is given in Table 3.

**Table 3.** Comparison Between Pediatric and Adult Artificial Intelligence–Enabled Devices

Characteristic	Devices, No. (%)^a^				
	All devices	Silent on age	Specific adult ages (≥18 y)	Generic all-ages labels	Specific pediatric ages (0-17 y)
Total	952 (100)	565 (59.3)	272 (28.6)	102 (10.7)	42 (4.4)
Year of oldest marketed device^b^	1997	1997	2008	2014	2015
Review time, median (IQR), d	135 (87-216)	134 (84-222)	158 (104-245)	107 (77-144)	162 (114-228)
Unique clinical areas	18 (100)	16 (88.9)	12 (66.7)	4 (22.2)	8 (44.4)
Radiology	723 (75.9)	457 (80.9)	164 (60.3)	93 (91.2)	18 (42.9)
Cardiovascular	99 (10.4)	37 (6.5)	55 (20.2)	6 (5.9)	4 (9.5)
Neurologic	34 (3.6)	11 (1.9)	20 (7.4)	0	13 (31.0)
With NCT	27 (2.8)	8 (1.4)	18 (6.6)	0	6 (14.3)
Recalls	136 (14.3)	32 (5.7)	11 (4.0)	32 (31.4)	5 (11.9)

Abbreviation: NCT, National Clinical Trial

^a^ *All devices* includes all entries in the Food and Drug Administration artificial intelligence–enabled devices database. *Silent on age* includes device indications with no mention of patient age. *Specific adult ages (≥18 y)* includes device indications encompassing adults or persons aged 18 years or older, including 35 technologies also indicated for pediatrics. *Generic all-ages labels* includes device indications mentioning persons of all ages (adults and pediatric populations) without specific age ranges listed. *Specific pediatric ages (0-17 y)* includes device indications encompassing persons aged 0 to 17 years, including 35 technologies also indicated for adults. *With NCT* includes device entries with at least 1 NCT number listed.

^b^ Excluding Papnet (initially approved in 1995), which is no longer marketed.

In addition to identifying devices specifically indicated for pediatric patients, we found 102 devices (10.7%) labeled for people of all ages or generically mentioning adult and pediatric applications. These technologies covered 4 of 18 clinical areas (22.2%), predominantly radiology (93 devices [91.2%]), and there were no clinical trial numbers associated with them to verify whether pediatric patients were included in device validation. The characteristics and complexity of these devices mostly resembled those of devices identified as being *silent on age* rather than specifically pediatric (Table 3). In rare instances, in lieu of age ranges (3 devices) or to supplement them (3 devices), physiologic descriptors (eg, >10 kg and 110 cm or ≥12 kg) were used to better define patient populations. We identified 7 sponsors (1.9%) with devices initially developed for *adults *or marked as *silent on age* who submitted applications for the labeling expansion to include pediatric age ranges (Figure 2). Although not recognized by the FDA as pediatric, 2 devices (0.2%) were explicitly labeled as indicated for fetal health applications.

## Discussion

This cross-sectional study provides an analysis of AI-enabled devices marketed in the US over the past 30 years, with a particular focus on their usability and applicability to pediatric patients. AI devices play a pivotal role in health care, especially in disease diagnosis, due to providing high diagnostic accuracy or serving as a clinical decision–support tool that improves productivity and confidence.^25,26^ Nevertheless, these benefits are largely reserved for adult populations given that little is known about the availability and use of AI technologies in pediatrics. We found that only 4.4% of all AI devices were specifically labeled for patients ages 0 to 17 years. We and others published extensively on the barriers in pediatric medical device innovation, the issue of off-label use of adult devices in children, and that pediatric devices lag in complexity and sophistication behind adult versions by as much as a decade.^8,15,16,17,18,19,20,21,22^ These issues appear to extend to the AI device space, as well; until 2015, there were no devices specifically indicated for pediatric patients, with the first exclusively pediatric device (Medo Aria) marketed in 2020, and only 7 sponsors (1.9%) sponsors expanded their initial device indications to include children and adolescents. Our comparative analysis revealed that pediatric devices covered only 8 of 18 clinical areas represented in the dataset. Interestingly, while some clinical areas were proportionally represented (eg, 10.4% of all devices vs 9.5% of devices labeled for individuals younger than 18 years were cardiovascular), other areas varied significantly (eg, neurology: 3.6% vs 31.0%). This may be due to AI innovation targeting conditions that are of particular relevance to pediatric populations or diagnosed early, including autism spectrum disorders, concussions, and epilepsy.

In addition to numerous barriers already limiting pediatric device innovation, the nature of AI technologies introduces several other unique challenges. First, is the access to robust pediatric datasets to enable AI training and validation. Data veracity requirements for AI development are considerable, yet high-quality datasets are necessary to minimize bias and maximize generalizability.^27,28,29^ This is particularly important for numerous image-based diagnostic applications, often in radiology and cardiology. These imaging methods’ low signal-to-noise ratios (eg, x-rays) require large datasets for AI training, while clinicians are cognizant of radiation exposure risks and the low diagnostic value of the method (depending on the clinical indication) and so may limit use of these methods (eg, x-rays) in pediatric patients. Improving diagnosis accuracy using AI has been studied and documented extensively, with numerous successful applications,^1,30,31,32^ but technical and clinical challenges remain and are a likely contributor to why AI pediatric devices in radiology and cardiology appear underrepresented compared with adult or all devices (Table 3). Data limitations are likely to be amplified for generative AI technologies, like large language models (LLMs), which demand larger, more diverse datasets and present additional evaluation challenges (eg, prompt sensitivity, output variability, and update cadence). For example, the NeoCLIP research program assembled more than 20 000 neonatal chest radiographs paired with clinical notes across 16 years to develop and evaluate a neonatal imaging model.^33^ As of this writing, no LLM-based devices have been authorized by FDA. While the agency has issued broad AI-enabled device guidance and finalized recommendations to support iterative updates, LLM-specific regulatory expectations for clinical use have not been published.^12,34,35^

The second likely factor is that medical device labeling regulations (21 CFR 801), unlike those for drugs (21 CFR 201), do not contain any pediatric considerations or age labeling instructions in general. To further convolute the issue, FDA CDRH defines pediatric patients as persons ages 0 to younger than 22 years for devices,^24^ while drug-specific federal regulations and the FDA Center for Drug Evaluation and Research define pediatric patients as those aged 0 to 16 years. Separate federal research regulations, under 45 CFR 46.402(a), define *children* as persons who have not attained the legal age for consent to treatments or procedures involved in the research; this is state dependent and could be younger than 18, 19, or 21 years. Notably, we found that only 12.9% of AI devices indicated for persons aged 0 to younger than 22 years were in alignment with FDA-recommended age ranges for pediatric subpopulations, while most devices (63.8%) were indicated for persons aged 18 years or older and 6 required the patient to be aged 20 or 21 years. These emerging patterns suggest that age labeling practices are inconsistent. Given that medical device regulations do not offer age-specific labeling standards, the interpretation of the intended patient population remains challenging.

Lastly, given that the safety of pediatric patients is paramount, numerous device manufacturers may be prioritizing testing in adults before including pediatric patients. Based on FDA data, only 27 device entries (2.8%) listed NCT numbers of their registered clinical trials. This rate was 14.3% for pediatric devices and 6.6% for devices specifically indicated for adults. Reporting of NCT numbers in device summaries is imperfect; nevertheless, if these rates approximate the use of clinical trials for device validation, the higher rate among pediatric-labeled devices suggests that reviewers may expect more pediatric-specific data. The FDA does not have distinct statutory evidentiary standards for pediatric devices; rather, review teams have latitude to make case-by-case benefit-risk determinations while adhering to least burdensome principles.^36,37^ In practice, greater evidence expectations intended to mitigate pediatric risks can increase development costs and lengthen timelines, potentially discouraging investment in pediatric innovation and leaving clinicians with off-label or less well-validated alternatives.

Simultaneously, a critical need exists to improve and adapt clinical trial infrastructure to the increasing demands for robust training data. This could include establishing pediatric medical device clinical trial units at children’s hospitals though the FDA Pediatric Device Consortia Program or a nationwide clinical trials network, such as one proposed though the Pediatric Medical Device Public Private Partnership.^38^ Future analysis of the clinical evidence used to support AI regulatory marketing decisions is required to inform FDA data demands. This is also needed to assess whether pediatric patients (or at least pediatric data) are being included in the AI training and validation datasets or in clinical studies evaluating AI performance.

A practical policy agenda could help address some of these challenges. First, the FDA should standardize device age labeling with structured data fields.^8,14,23,38^ When devices are marketed for *all ages*, the FDA should require or strongly encourage pediatric performance reporting, or at a minimum a strong justification of why it is not necessary. Pediatric-focused predetermined change control plans that allow iterative model updates with targeted postmarket commitments can support labeling expansion when premarket pediatric data are limited. Investing in shared pediatric datasets through federal and consortium mechanisms that support privacy-preserving linkage and access can lower development costs. Addressing financial and operational barriers to observational studies can lead to more and faster dataset creation Finally, harmonizing pediatric definitions across FDA centers can reduce ambiguity for sponsors and reviewers.

### Limitations

This cross-sectional analysis has several limitations, including that it relies on publicly available FDA resources, such as the AI-enabled medical devices list and FDA medical device databases, which are imperfect and sometimes incomplete. As the FDA acknowledges, the list is not exhaustive and its curation methods are not fully described; the list was analyzed as accessed in November 2024, but subsequent versions include additional listings. Information within FDA decision summaries and indications for use (eg, age ranges, clinical evidence, and trial registration) is variably reported, which can lead to misclassification of pediatric labeling or clinical area. Although we combined manual abstraction with natural language processing to extract variables, errors may have been introduced at either stage despite quality checks. We were unable to consistently ascertain algorithmic characteristics (eg, model type, training data sources, or update cadence) across devices, limiting inferences about the kinds of AI technology used and their pediatric performance. Because these data reflect marketed devices and publicly reported documentation rather than full regulatory dossiers, we cannot determine the totality of evidence considered by FDA reviewers or the causal determinants of pediatric labeling. Additionally, the analysis is limited to US regulatory databases given that comparable databases do not exist in other jurisdictions.

## Conclusions

This cross-sectional study found that pediatric labeling among FDA-marketed AI-enabled devices was rare, recent, and concentrated in a few clinical areas, with frequent ambiguity around age indications. These patterns, along with inconsistent publicly available data, limit clinician ability to assess pediatric applicability and may slow or inhibit access to AI-supported care for pediatric patients. Policy changes addressing device labeling, evidence requirements, and pediatric-specific considerations could reduce friction, alongside investments in pediatric clinical research infrastructure and dataset development. Taken together, these pragmatic steps could support the safe and effective deployment of AI technologies for pediatric patients of all ages.

## Notes

Supplement 1. **eTable.** Characteristics of Artificial Intelligence–Enabled Device Sponsors

Supplement 2.
Data Sharing Statement.
