# Supplementary material for: FDA-Regulated AI-Enabled Medical Devices With Pediatric Indications
Source: JAMA Netw Open. 2026 Mar 20;9(3):e262636. doi: 10.1001/jamanetworkopen.2026.2636 (PMC13005162; doi:10.1001/jamanetworkopen.2026.2636)
Supplement: Supplement 1. — eTable. Characteristics of Artificial Intelligence–Enabled Device Sponsors [file jamanetwopen-e262636-s001.pdf]

Supplemental Online Content

Zapotoczny G, Goyal A, Christmas M, Qazi S, Carroll M, Espinoza J. FDA-regulated AI-enabled medical devices with pediatric indications. *JAMA Netw Open*. 2026;9(3):e262636. doi:10.1001/jamanetworkopen.2026.2636

eTable. Characteristics of Artificial Intelligence–Enabled Device Sponsors

This supplemental material has been provided by the authors to give readers additional information about their work.

© 2026 Zapotoczny G et al. *JAMA Network Open*.

eTable. Characteristics of Artificial Intelligence–Enabled Device Sponsors

Characteristic	Sponsors, No. (%)	Corresponding devices, No. (%)
All sponsors	372 (100)	952 (100)
With >20 devices	6 (1.6)	233 (24.47)
With <5 devices	335 (90.1)	499 (52.4)
With 1 device	228 (61.3)	228 (23.9)
International (29 countries)	190 (51.1)	485 (50.9)
Israel (top 1 country)	29 (7.8)	77 (8.1)
Domestic (33 states)	182 (48.9)	467 (49.1)
California (top 1 state)	60 (16.1)	117 (12.3)

© 2026 Zapotoczny G et al. *JAMA Network Open*.
