# Supplementary material for: FDA-Regulated AI-Enabled Medical Devices With Pediatric Indications
Source: JAMA Netw Open. 2026 Mar 20;9(3):e262636. doi: 10.1001/jamanetworkopen.2026.2636 (PMC13005162; doi:10.1001/jamanetworkopen.2026.2636)
Supplement: Supplement 2. — Data Sharing Statement. [file jamanetwopen-e262636-s002.pdf]

**Data Sharing Statement**

Zapotoczny. FDA-Regulated AI-Enabled Medical Devices With Pediatric Indications. *JAMA Netw Open*. Published online March 20, 2026. doi:10.1001/jamanetworkopen.2026.2636

**Data**
**Data available:** Yes
**Data types:** Data (not involving human participants)
**How to access data:** Data available upon request: jespinozasalomon@luriechildrens.org
**When available:** With publication

**Supporting Documents**
**Document types:** None

**Additional Information**
**Who can access the data:** anyone requesting the data
**Types of analyses:** any purpose
**Mechanisms of data availability:** website link
